# Pacing-Induced Cardiomyopathy in a Transplanted Heart Treated With Left Bundle Branch Pacing

**DOI:** 10.1016/j.jaccas.2025.105537

**Published:** 2025-09-25

**Authors:** Nicholas Tomassoni, Roy Sun, Marc Erickson, Pugazhendhi Vijayaraman

**Affiliations:** a Geisinger Commonwealth School of Medicine, Scranton, Pennsylvania, USA; b Geisinger Heart Institute, Geisinger Wyoming Valley Medical Center, Wilkes-Barre, Pennsylvania, USA

**Keywords:** cardiac pacemaker, cardiac resynchronization therapy, cardiac transplant, chronic heart failure, left bundle branch pacing

## Abstract

**Background:**

Atrioventricular (AV) block is a potential complication of orthotropic heart transplantation (OHT). The optimal cardiac pacing method is largely unexplored in patients after OHT.

**Case Summary:**

We present a patient with AV block after OHT in whom right ventricular pacing resulted in pacing-induced cardiomyopathy (PICM). Left ventricular function was restored with left bundle branch pacing.

**Discussion:**

AV block is an uncommon complication after OHT that requires ventricular pacing. Ventricular dyssynchrony induced by right ventricular pacing may be associated with increased risk for PICM in OHT recipients. Cardiac physiologic pacing should be considered as the primary pacing strategy in this population.

**Take-Home Messages:**

OHT recipients may be at increased risk for PICM. Left bundle branch pacing is an effective strategy for both prevention and treatment of PICM.

## Graphical Abstract

**Figure:**
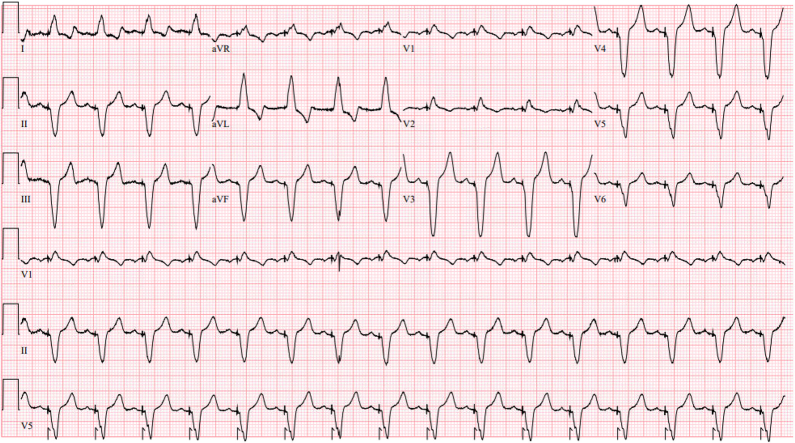

## History of Presentation

A 73-year-old man with a history of severe left ventricular systolic dysfunction, ischemic cardiomyopathy, and ventricular tachycardia, with subsequent orthotropic heart transplant (OHT) in 2021, received permanent dual-chamber pacemaker for complete atrioventricular (AV) block that developed immediately after transplantation. Leads were placed in the right atrial appendage and the right ventricular apex.

**Take-Home Messages**

• AV block after heart transplant is not uncommon.
• Heart transplant recipients may be at increased risk for adverse outcomes from right ventricular pacing.
• LBBP is an effective therapeutic approach to reverse PICM in a transplanted heart.
• LBBP should be considered as a primary pacing modality in heart transplant recipients requiring ventricular pacing.

On presentation in 2023, the patient had a body mass index of 29.35 kg/m^2^, blood pressure of 118/60, and heart rate of 97 beats/min. Electrocardiogram showed atrial sensed right ventricular pacing at 100 beats/min, QRS duration of 168 ms, and QTc of 541 ms. Ventricular pacing was at 100%. His NYHA functional status had declined to class II, with exertional dyspnea and mild leg edema. Routine echocardiogram revealed a decline in left ventricular ejection fraction to between 40% and 45%.

## Medical History

The patient's medical history included: 1) Graves disease; 2) post–radioiodine therapy hypothyroidism; 3) myocardial infarction; 4) coronary artery disease; 5) paroxysmal ventricular tachycardia; 6) AV nodal re-entrant tachycardia; 7) thrombocytopenia; and 8) OHT.

In June 2021, the patient underwent bicaval OHT for management of chronic systolic heart failure, prior myocardial infarction, ventricular tachyarrhythmias requiring multiple defibrillator shocks, and atrial fibrillation. Before his OHT, he had undergone successful atrial fibrillation ablation and palliative ventricular tachycardia ablation. The left ventricular ejection fraction in the transplanted heart was 65% to 70%. The patient was then placed on dual immunosuppressive agents.

After transplant, the patient experienced complete heart block with sinus node dysfunction. A permanent dual-chamber pacemaker was placed 1 week post-transplant. Sinus node function gradually recovered, and follow-up evaluation demonstrated minimal atrial pacing and 100% ventricular pacing. The patient recovered well and improved from NYHA functional class III pretransplant to class I within 6 months post-transplant.

## Differential Diagnosis

Differential diagnosis of new-onset left ventricular (LV) dysfunction in the transplanted heart included: 1) cardiac allograft vasculopathy; 2) graft rejection; and 3) right ventricular pacing-induced cardiomyopathy (PICM).

## Investigations

**Figure 1:**
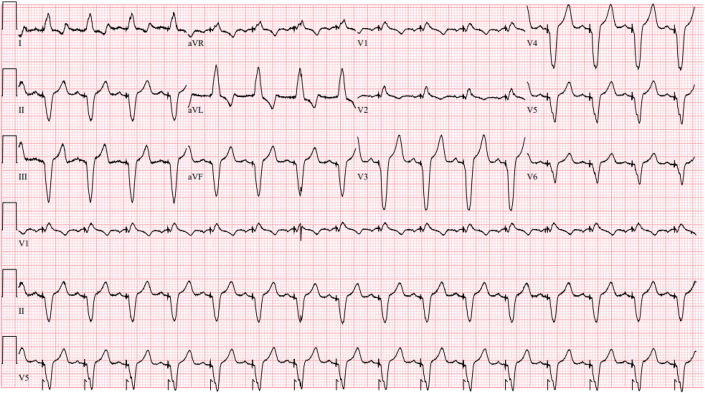
Electrocardiogram 17 Months After Orthotropic Heart Transplantation Demonstrates Atrial Sensed Right Ventricular Apical Pacing

Cardiac allograft vasculopathy and rejection were ruled out by coronary angiography and endomyocardial biopsy. Thyroid-stimulating hormone and T4 levels remained normal. Electrocardiogram taken during this period (Figure 1) revealed QRS prolongation (170 ms) and left-axis deviation (−70°). Pacemaker interrogation showed 100% pacing in the right ventricle.

## Management

**Figure 2:**
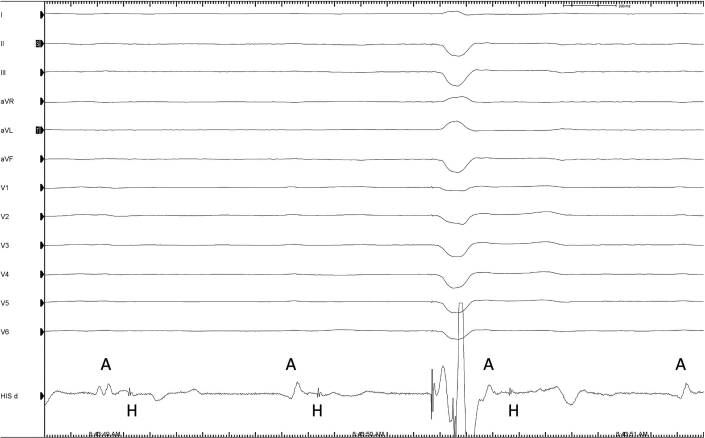
Electrograms at the His-Bundle Region Shows Atrial Signals Followed by His-Bundle Potentials Without Conduction to the Ventricle, Confirming His-Ventricular Block Atrial electrogram shows His-bundle potentials without conduction to the ventricle, confirming His-ventricular block.

Considering right ventricular pacing (RVP)–induced ventricular dyssynchrony as the etiology of his new-onset LV systolic dysfunction, the patient was referred for cardiac resynchronization therapy (CRT). After discussing options of CRT using biventricular pacing with coronary sinus pacing versus conduction system pacing (CSP) with left bundle branch pacing (LBBP), a shared decision was made to proceed with CRT upgrade with LBBP. During the procedure, His-bundle mapping was performed using a Medtronic C315His sheath and Medtronic SelectSecure 3830 pacing lead. Complete His-ventricular block was demonstrated (Figure 2).

**Figure 3:**
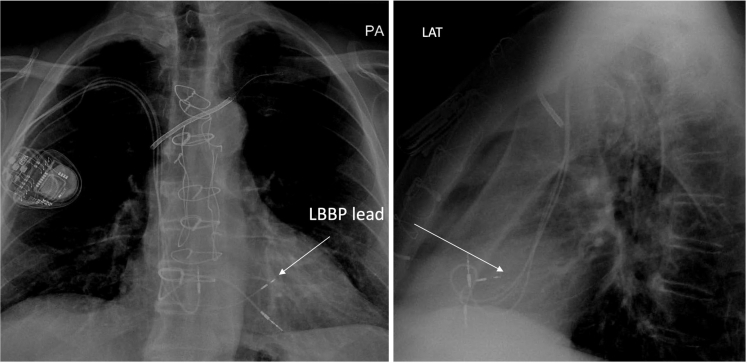
Posteroanterior and Lateral Chest X-Rays of the Left Bundle Branch Pacing Lead Location (Arrow)

The sheath and the lead were advanced 2 cm distal to the His into the right ventricle. The lead was then advanced approximately 14 mm deep into the interventricular septum (septal thickness: 13 mm) until the LV endocardium was reached. Pacing from this location confirmed left bundle branch capture (transition during threshold testing, R-wave peak time in V6 of 78 ms). The new LBBP lead was connected to the LV port of a CRT-P device while retaining the right atrial and right ventricular pacing leads. An LV-RV delay of 80 ms was programmed to achieve LV-only pacing. Chest X-ray images of the lead location are shown in Figure 3. The patient recovered without complications.

## Outcome and Follow-Up

**Figure 4:**
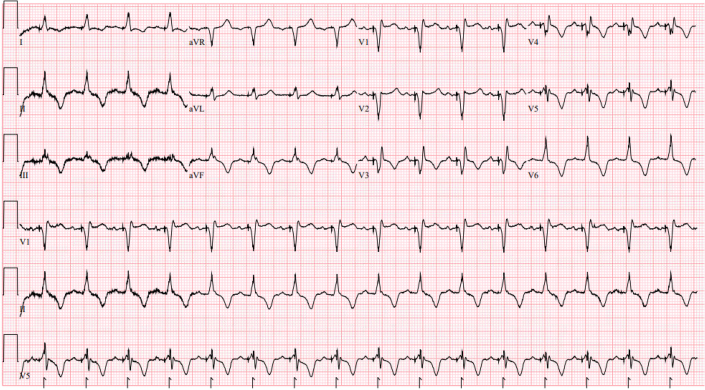
Electrocardiogram After the Procedure Electrocardiogram shows injury current, right bundle branch block pattern, and memory T waves in the precordial leads.

**Figure 5:**
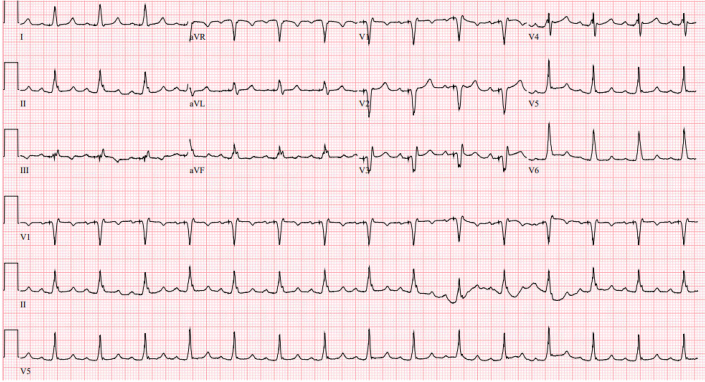
Electrocardiogram at 3 Months After Upgrade The QRS remains relatively narrow, and T waves have returned to their previous morphology.

Immediately after pacemaker upgrade, the patient's surface electrocardiogram exhibited narrowing of paced QRS duration (138 ms), with minimal right ventricular conduction delay pattern and cardiac memory T-wave changes (ie, inverted T waves on precordial and limb leads) (Figure 4).^1^ The inverted T waves completely normalized at 3 months postprocedure, further indicating recovery of normal ventricular depolarization and repolarization (Figure 5).

**Figure 6:**
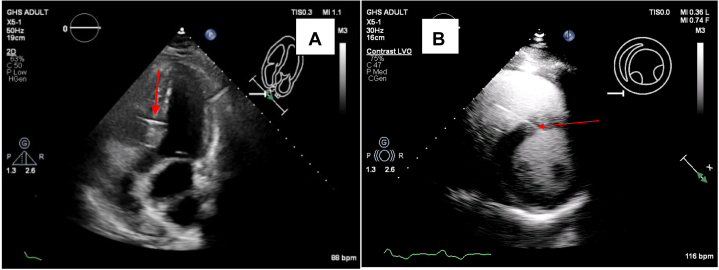
Echocardiogram at 3 Months After Upgrade (A) Long-axis apical and (B) short-axis views of the patient's heart on echocardiogram. The pacing lead (red arrows) is buried securely in the left ventricular septal subendocardium.

An echocardiogram was performed at 3 months postprocedure to verify the placement and stability of the new pacing lead and to quantify cardiac function (Figure 6). The LV ejection fraction increased to between 60% and 64%, which the patient presently maintains. The absolute global longitudinal peak systolic strain improved from 12.2% to 30.6%. The patient has remained symptom free.

## Discussion

Cardiac conduction abnormalities after OHT are not uncommon. The etiology of these conduction abnormalities includes advanced donor age, biatrial anastomosis, prolonged ischemic times, graft failure, and acute rejection.^2^ Both sinus node dysfunction and AV conduction abnormalities can occur after OHT.^3^ Our patient experienced complete AV block post-transplant, with early sinus node dysfunction requiring permanent pacing. In a recent study, early pacemaker dependency in OHT recipients was associated with increased risk for permanent pacemaker and subsequent increased risk of mortality compared with patients who did not have pacemaker dependency in the postoperative period.^4^

Our patient received a traditional dual-chamber pacemaker with right ventricular apical pacing after his OHT. During follow-up, he developed significant cardiac dyssynchrony from RVP and mild LV systolic dysfunction. In the absence of graft rejection or vasculopathy, combined with long-term dependence of 100% RVP, he was diagnosed with PICM.^5^

Compared with RVP, CSP using His bundle pacing or left bundle branch pacing has been shown to reduce the adverse clinical outcomes of heart failure hospitalization, mortality, or need for subsequent upgrade to biventricular pacing.^6^^,^^7^ The exact incidence of PICM in heart transplantation recipients is unknown. It is unclear if patients requiring permanent ventricular pacing after heart transplant should routinely undergo biventricular pacing or CSP. His bundle pacing or LBBP has been reported in patients requiring pacing or CRT several years after OHT.^8^, ^9^, ^10^, ^11^ LBBP was recently reported in a patient with mild LV dysfunction and second-degree AV block few days after OHT, with subsequent improvement of LV ejection fraction.^12^ CSP has been shown to reverse PICM in native hearts.^13^^,^^14^ Our patient successfully underwent LBBP, with normalization of LV function. Interestingly, His-ventricular block was demonstrated to be the primary conduction abnormality. The exact pathophysiology of block in the His bundle in this patient after OHT is unclear.

Given the possibility of conduction disturbances after OHT and the potential risk for PICM, we suggest consideration of LBBP as the primary pacing modality to prevent PICM.

## Conclusions

The present case highlights the potential development of PICM in OHT recipients. Heart transplant patients who receive RVP should be monitored for PICM. Physiological pacing strategies such as LBBP may reverse the PICM in heart transplant patients.

## Funding Support and Author Disclosures

Dr Vijayaraman has received honoraria, consultant fees, research support, and fellowship support from Medtronic; has received consultant fees and/or honoraria from Abbott and Biotronik; and has a patent with Boston Scientific. All other authors have reported that they have no relationships relevant to the contents of this paper to disclose.
